# Risk prediction of second primary malignancies in patients after rectal cancer: analysis based on SEER Program

**DOI:** 10.1186/s12876-023-02974-2

**Published:** 2023-10-12

**Authors:** Yong-Chao Sun, Zi-Dan Zhao, Na Yao, Yu-Wen Jiao, Jia-Wen Zhang, Yue Fu, Wei-Hai Shi

**Affiliations:** 1 Graduate School of Bengbu Medical College, Anhui, China; 2 Department of General Surgery, The Affiliated Changzhou No. 2 People’s Hospital of Nanjing Medical University, Changzhou, 213003 Jiangsu China; 3 Department of Breast Surgery, The Affiliated Wuxi Hospital of Nanjing University of TCM, Wuxi City Hospital of TCM, Wuxi, China

**Keywords:** Seer, Rectal cancer, Second primary malignancy, Nomogram, Treatment decision

## Abstract

**Background:**

This study will focus on exploring the clinical characteristics of rectal cancer (RC) patients with Second Primary Malignancies (SPMs) and constructing a prognostic nomogram to provide clinical treatment decisions.

**Methods:**

We determined the association between risk factors and overall survival (OS) while establishing a nomogram to forecast the further OS status of these patients via Cox regression analysis. Finally, we evaluated the performance of the prognostic nomogram to predict further OS status.

**Results:**

Nine parameters were identified to establish the prognostic nomogram in this study, and, the C-index of the training set and validation set was 0.691 (95%CI, 0.662–0.720) and 0.731 (95%CI, 0.676–0.786), respectively. The calibration curve showed a high agreement between the predicted and actual results, and the receiver operating characteristic (ROC) curves verified the superiority of our model for clinical usefulness. In addition, the nomogram classification could more precisely differentiate risk subgroups and improved the discrimination of SPMs’ prognosis.

**Conclusions:**

We systematically explored the clinical characteristics of SPMs after RC and constructed a satisfactory nomogram.

**Supplementary Information:**

The online version contains supplementary material available at 10.1186/s12876-023-02974-2.

## Introduction

Rectal cancer represents the eighth most frequent diagnosed malignancy and the tenth most common reason for cancer-related deaths globally in 2018, [1] with approximately 732,210 new cases and 339,022 fatalities in 2020 [2, 3]. Nowadays, due to the progress of early diagnosis, comprehensive treatment, and advances in cancer detection, the OS of RC patients has greatly improved [4]. For early-treated rectal cancer, the 5-year OS rate among patients could even reach 90% [5, 6]. However, second primary malignancies are threatening the lives of RC patients who underwent long-term survival [7]. Recently, A growing number of studies have been carried out to investigate the risk factors for the development of SPMs in specific tumors, such as lung cancer [8], prostate cancer [9], breast cancer [10], stomach cancer [11], and so on. The prevalence of SPMs in RC survivors has been reported in earlier studies is 4-8% higher than in the normal population [12]. Factors thought to be influencing this higher rate have been explored in several studies, related to the patient’s genetic factors, lifestyle, environmental risk factors, and cancer therapy [13–15].

Nomogram have been identified as a simpler and more sophisticated clinical prediction tool for predicting individualized OS based on clinical characteristics and risk factors [16–18]. We discover that it is extremely important to understand the incidence and prognosis of SPM patients for treatment providers and RC patients. Therefore, this study will concentrate on the risk factors for SPMs and will develop a nomogram to forecast the 1-, 3-, and 5-year OS of SPMs after RC.

## Materials and methods

### Data source

Methods Data were obtained from SEER Research Plus Date,18 Registries, Nov 2020 Sub(2000–2018) in the Surveillance, Epidemiology, and End Results (SEER) database(http:/ /seer.cancer.gov)using SEER* Stat version 8.4.0. Clinicopathological information was gathered including age, race, gender, SPMs site, tumor size, histological type of SPMs and RC, TNM stage, clinical stage, surgical history of SPMs and RC, chemotherapy, radiotherapy, marital status, follow-up time, latency between RC and SPMs, respectively.

### Definition of SPMs

SPMs was defined as metachronous invasive solid cancer developing ≥ 6 months after initial primary cancer (IPC), under criteria of Warren and Gates as modified by the National Cancer Institute [19]. The SEER database listed the pathologic subtypes of IPC and SPMs. To better distinguish SPMs from primary and metastatic tumors, we defined SPMs as second malignancy and histological different from IPC with an incubation period of not less than 6 months. Likewise, SEER database provided key clinical information on “malignant tumors for patient” and the “sequence number” of the multiple primary malignancies. It could be used to identify patients with SPM and index the sequence of multiple malignancies.

### Patient selection

**Fig. 1:**
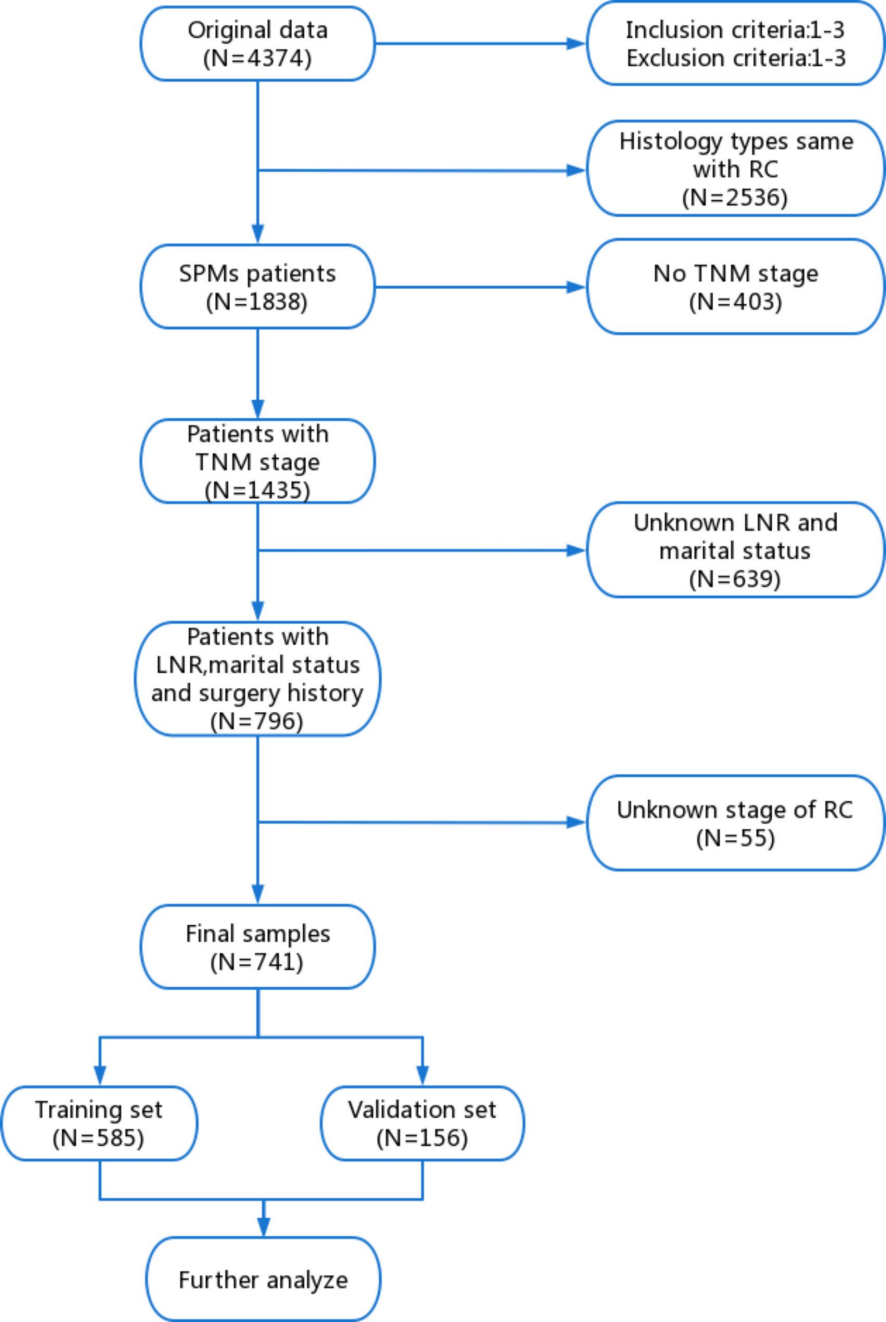
Study flowchart showing the process of constructing nomogram to predict the overall survival (OS) of second primary malignancies (SPMs) after rectal cancer (RC). LNR: lymph node removed

The clinicopathological information of a total of 4374 patients with rectal cancer was obtained from the SEER database. The following were the inclusion criteria: (1) Diagnosed age was between 20 and 80 years. (2) Rectal cancer was discovered in patients between January 2004 and December 2013, and the follow-up period was at least 5 years; (3) Detailed survival data and follow-up information on patients should be provided. The following were the exclusion criteria: (1) Patients without pathological confirmation of the diagnosis; (2) Patients who only provided death certificate records or autopsy records; (3) Latency periods of fewer than 6 months between IPC and SPMs. Next, we screened for the same histological type as rectal cancer (N = 2536), wherein 1838 patients were still diagnosed with SPMs. Patients with unclear clinical data were excluded, including the patients who have no TNM stage (N = 403), unknown lymph node removed (LNR) and marital status (N = 639), and unknown clinical stage of RC (N = 55). Finally, the prognostic nomogram was created using the risk factors that were identified, which were integrated from the detailed clinical data of 741 SPM patients with rectal cancer. Then, the data of 741 patients were randomly split into a training set (N = 585) and a verification set (N = 156) at a ratio of 7:2. Meanwhile, the training and validation set were used for external and internal validation, respectively. The precise details of SPMs screening were shown in Fig. 1.

### Statistical analysis

To investigated the relationship between clinicopathological variables and OS of SPMs, univariate and multivariate Cox regression analyses were performed to specify the risk factors. Next, significantly different risk factors were used to build a nomogram that accurately forecast the 1-, 3- and 5-year survival rates of SPM patients. To verified the performance of the nomogram we constructed, the C-index was used to assess the accuracy of the prediction results. Next, the calibration curve was created to evaluate the consistency between predicted and actual results while bootstrapping with 1000 resamples was used to assess discrimination and calibration. Then, survival predictions for 1-, 3-and 5-year were estimated using the ROC curve. In addition, the nutrition risk index (NRI) and integrated discrimination improvement (IDI) were used to evaluate the degree the of accuracy between the nomogram and the conventional AJCC staging system, And the clinical usefulness and benefits of the nomogram were estimated by the decision curve analysis (DCA) plots.

In this study, R software (version 4.1.2) and SPSS 25.0 were both used for all statistical analysis. All tests were two-way and *P* < 0.05 was considered statistically significant.

## Results

### Characteristics of patients

**Fig. 2:**
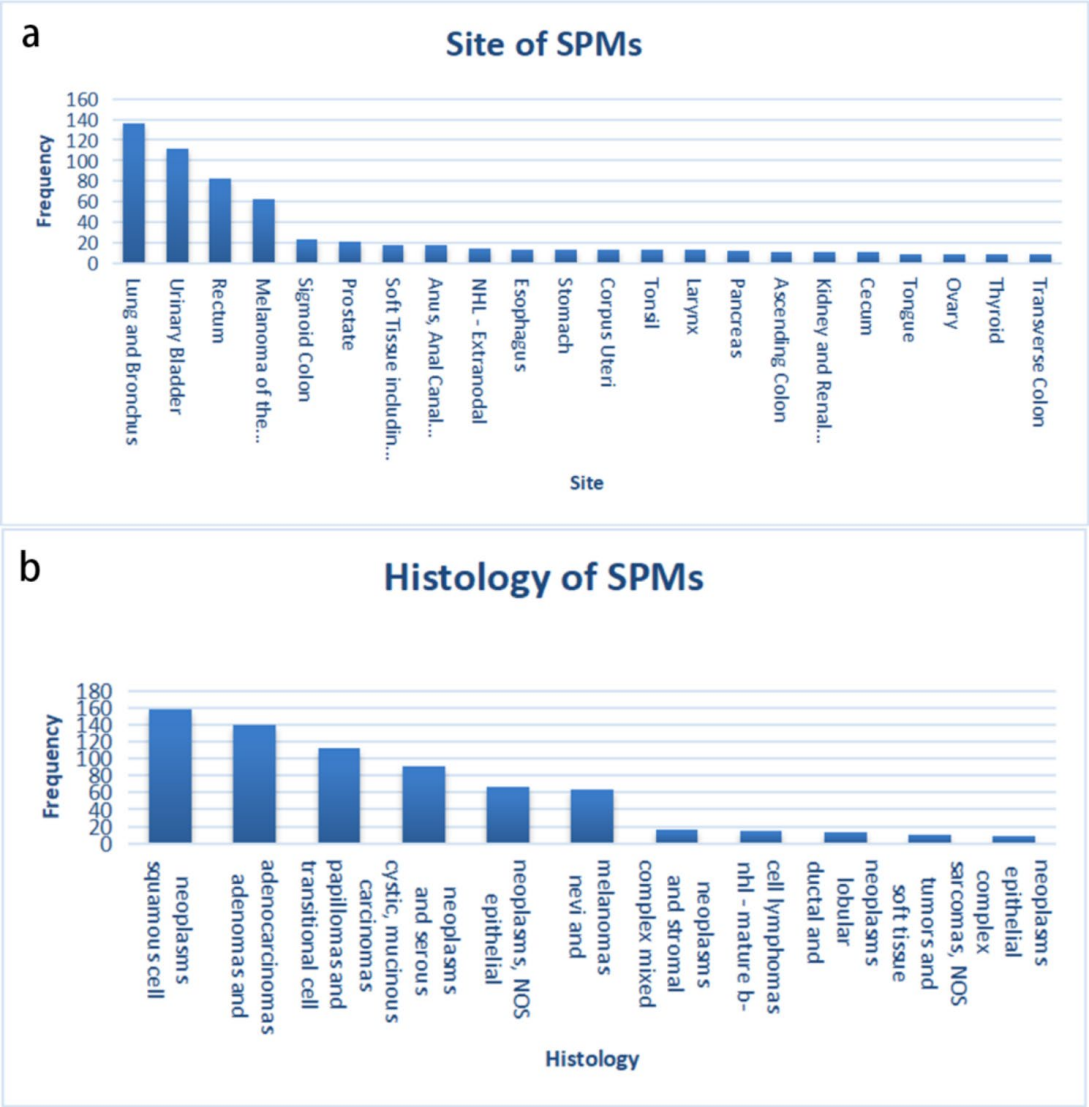
Features of second primary malignancies (SPMs) after rectal cancer (RC). **(a)** Sites of SPMs that over than 1%, **(b)** Histology types of SPMs that more than 1%

A total of 51,611 patients diagnosed with rectal cancer during 2004–2013 was obtained from the SEER database, of which 4,374 patients were diagnosed with cancer more than 6 months after the initial diagnosis of RC. To rule out caused recurrence and metastasis of RC, the patient’s data with the same histological type as RC was ruled out. Ultimately, a total of 1838 (3.56%) patients diagnosed with SPMs were identified. The results showed that the median interval between RC and SPMs diagnosis was 36 months and the median age at SPMs diagnosis was 67.5 years. By using original data obtained from the SEER database, 741 cases of SPMs were found. After removing those with unclear clinical information, more than 1% of the patients’ SPM sites and histological types were listed (Fig. 2), suggesting that the three most common sites for SPMs were the Lung and Bronchus (18.35%), Urinary Bladder (15.11%), and Breast (11.20%) (Table 1) (Table S1). The three most prevalent histological types for SPMs were Squamous Cell Neoplasms (21.32%), Adenomas and Adenocarcinomas (18.76%), Transitional Cell Papillomas and Carcinomas (15.11%) (Table 1) (Table S2).

**Table 1:** Site and Histology types of SPMs after RC that the top 20

Site of SPMs	N	%	Histology of SPMs	N	%
All	741	100.00%	ALL	741	100.00%
Lung and Bronchus	136	18.35%	Squamous Cell Neoplasms	158	21.32%
Urinary Bladder	112	15.11%	Adenomas and Adenocarcinomas	139	18.76%
Rectum	83	11.20%	Transitional Cell Papillomas and Carcinomas	112	15.11%
Melanoma of the Skin	63	8.50%	Cystic, Mucinous and Serous Neoplasms	91	12.28%
Sigmoid Colon	23	3.10%	Epithelial Neoplasms, NOS	67	9.04%
Prostate	21	2.83%	Nevi and Melanomas	64	8.64%
Soft Tissue including Heart	18	2.43%	Complex Mixed and Stromal Neoplasms	16	2.16%
Anus, Anal Canal and Anorectum	18	2.43%	Nhl - Mature B-Cell Lymphomas	14	1.89%
NHL - Extranodal	15	2.02%	Ductal and Lobular Neoplasms	13	1.75%
Esophagus	14	1.89%	Soft Tissue Tumors and Sarcomas, NOS	10	1.35%
Stomach	14	1.89%	Complex Epithelial Neoplasms	8	1.08%
Corpus Uteri	14	1.89%	Fibromatous Neoplasms	7	0.94%
Tonsil	13	1.75%	Acinar Cell Neoplasms	7	0.94%
Larynx	13	1.75%	Germ Cell Neoplasms	6	0.81%
Pancreas	12	1.62%	Oseous and Chondromatous Neoplasms	3	0.40%
Ascending Colon	11	1.48%	Lipomatous Neoplasms	3	0.40%
Kidney and Renal Pelvis	11	1.48%	Myomatous Neoplasms	3	0.40%
Cecum	11	1.48%	Basal Cell Neoplasms	3	0.40%
Tongue	9	1.21%	Mesothelial Neoplasms	3	0.40%
Ovary	9	1.21%	Nhl - Mature t and Nk-Cell Lymphomas	2	0.27%

Abbreviations: SPMs: second primary malignancies; RC: rectal cancer

Final enrollment for further analysis included 741 patients in total, both the training set (N = 585) and the validation set (N = 156) were randomly divided from the 741 patients. Meanwhile, there was no significant difference in clinical information by using the χ2 test (*P* > 0.05), including the site of SPMs, histology of SPMs, age, race, TNM stage, treatment information, tumor size, and grade of SPMs (Table 2). The training set was used to build the nomogram and verify the model internally, while the validation set was utilized for external validation.

**Table 2:** Clinicopathological characteristics of SPM patients with RC

Variables	Training set		Validation set			χ²	*P* value
	(n = 585)		(n = 156)				
	N	%	N	%			
Site of SPMs						0.75	0.980
Lung and Bronchus	106	18.1	30	19.2			
Urinary Bladder	88	8.7	24	7.7			
Rectum	68	43.4	15	44.9			
Melanoma of the Skin	51	11.6	12	9.6			
Sigmoid Colon	18	3.1	5	3.2			
Others	254	15.0	70	15.4			
Histology of SPMs						6.54	0.257
Squamous Cell Neoplasms	130	22.2	28	17.9			
Adenomas and Adenocarcinomas	114	19.5	25	16.0			
Transitional Cell Papilloma and Carcinomas	90	15.4	22	14.1			
Cystic, Mucinous and Serous Neoplasms	64	10.9	27	17.3			
Nevi and Melanomas	51	8.7	13	8.3			
Others	136	23.2	41	26.3			
Age(years)						0.79	0.853
<60	152	26.0	42	26.9			
60–69	190	32.5	47	30.1			
70–79	214	36.6	61	39.1			
≥ 80	29	5.0	6	3.8			
Race						5.29	0.071
White	484	82.7	127	81.4			
Black	65	11.1	12	7.7			
Others	36	6.2	17	10.9			
Stage-T						2.81	0.590
Ta	141	24.1	34	21.8			
T1	186	31.8	42	26.9			
T2	122	20.9	38	24.4			
T3	89	15.2	26	16.7			
T4	47	8.0	16	10.3			
Stage-N						5.15	0.161
N0	482	82.4	113	74.4			
N1	55	9.4	22	14.1			
N2	43	7.4	16	10.3			
N3	5	0.9	5	1.3			
Stage-M						0.29	0.593
M0	528	90.3	143	91.7			
M1	57	9.7	13	8.3			
Stage-T of RC						2.71	0.608
Ta	74	12.6	24	15.4			
T1	140	23.9	34	21.8			
T2	87	14.9	29	18.6			
T3	254	43.4	63	40.4			
T4	30	5.1	6	3.8			
Stage-N of RC						3.72	0.155
N0	437	74.7	106	67.9			
N1	110	18.8	34	21.8			
N2	38	6.5	16	10.3			
Stage-M of RC						0.91	0.341
M0	552	94.4	144	92.3			
M1	33	5.6	12	7.7			
SPMs Surgical history							
Yes	422	72.1	113	72.4		0.005	0.941
No	163	27.9	43	27.6			
Surgical history of RC						0.35	0.554
Yes	511	87.4	139	89.1			
No	74	12.6	17	10.9			
Histology of RC						0.75	0.689
Others	70	12.0	19	12.2			
Ade	436	74.5	120	76.9			
Cystic, Mucinous and Serous Neoplasms	79	13.5	17	10.9			
SPMs radiation record						0.58	0.445
Yes	151	25.8	45	28.8			
No	434	74.19	111	71.2			
Radiation record of RC						0.83	0.362
Yes	291	49.7	84	53.8			
No	294	50.3	72	46.2			
SPMs chemotherapy record						0.96	0.328
Yes	215	36.8	64	41.0			
No	370	63.2	92	59.0			
Chemotherapy record of RC						1.06	0.303
Yes	318	54.4	92	59.0			
No	267	45.6	64	41.0			
SPMs tumor size(cm)						5.45	0.141
0–3	394	67.4	97	62.2			
3–5	86	14.7	32	20.5			
5–10	78	13.3	16	10.3			
≥ 10	27	4.6	11	7.1			
SPMs grade						4.73	0.316
Well	56	9.6	9	5.8			
Moderately	183	31.3	41	26.3			
Poorly	94	16.1	29	18.6			
Undifferentiated	45	7.7	15	9.6			
Unknown	207	35.4	62	39.7			

Abbreviations: SPMs: second primary malignancies; RC: rectal cancer, Ade: adenomas and adenocarcinomas

### Prognostic factors selection and nomogram construction

**Fig. 3:**
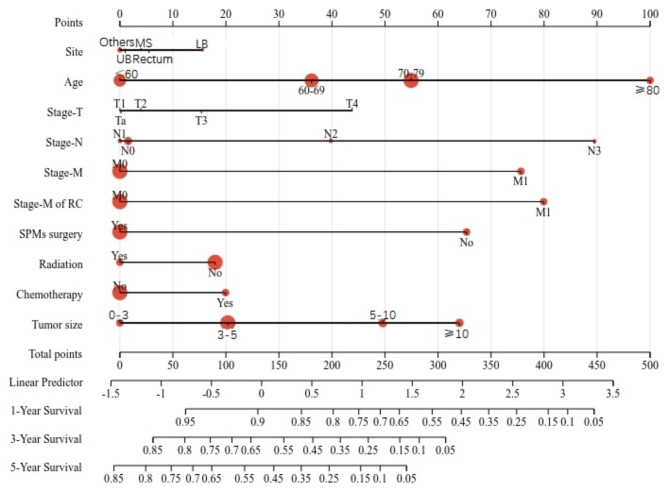
Nomogram to predict 1-,3- and 5-year survival for second primary malignancies (SPMs); MS: Lung and Bronchus; UB: Urinary Bladder; LB: Melanoma of the Skin; Others: Less than 5%

Univariate and multivariate Cox regression analysis was applied to reveal OS-related factors in SPMs. The results (Table 3) show that the OS of SPMs was a significantly higher risk with age, TNM stage, stage M of RC, SPMs surgical history, SPMs tumor size (*P* < 0.001) and site(*P* = 0.009), while the OS of SPMs was a significantly lower risk with chemotherapy and radiotherapy(*P*<0.001). Multivariate Cox regression analysis revealed that age, stage-M, stage-M of RC, and SPMs surgical history(*P*<0.001), stage-T(*P* = 0.003), and stage-N(*P* = 0.012) were independent predictive variables for SPMs survival. According to the results of univariate and multivariate Cox regression analysis, 9 parameters including the site, age, stage TNM, stage M of RC, SPMs surgical history, SPMs radiotherapy records, SPMs chemotherapy records, and SPMs tumor size were used to establish a nomogram for predicting 1-, 3-, and 5-year OS (Fig. 3). To use the nomogram more conveniently, each of these characteristics was allocated a particular point on the scale. A total point was received for the individual patients, followed by a summary of the points from each parameter. Then, the probability of OS occurrence after 1, 3, and 5 years was predicted by transferring the entire score to the nomogram’s total score table. As an example, the total point of all variables for an SPM patient diagnosed with 60 years in urinary bladder site of 5 cm Tumor size, T2N2M0, M0 of RC, having SPMs Surgery record and Radiation record, but no chemotherapy record was 135, which corresponded to 1-,3-, and 5- year OS rates of about 88.3%,62.5%, and 50.1%, respectively.

**Table 3:** Univariate and multivariate Cox analysis of SPMs patients after RC in the training and validation set

Variables	Univariate analysis			Multivariate analysis		
	HR	CI (95%)	*P* value	HR	CI (95%)	*P* value
Site of SPMs			0.009			0.108
Lung and Bronchus	1.000			1.000		
Urinary Bladder	0.383	0.276–0.530	<0.001	1.651	0.735–3.713	0.225
Rectum	0.507	0.366–0.701	<0.001	1.020	0.648–1.605	0.932
Melanoma of the Skin	0.349	0.229–0.530	<0.001	0.217	0.029–1.630	0.137
Sigmoid Colon	0.427	0.243–0.748	<0.001	0.854	0.450–1.621	0.629
Others	0.522	0.410–0.665	<0.001	0.840	0.619–1.140	0.263
Age(years)			<0.001			<0.001
<60	1.000			1.000		
60–69	1.431	1.099–1.863	0.008	1.422	1.074–1.883	0.014
70–79	1.758	1.370–2.256	<0.001	1.713	1.297–2.263	<0.001
≥ 80	2.499	1.624–3.846	<0.001	2.801	1.763–4.450	<0.001
Stage-T			<0.001			0.003
Ta	1.000			1.000		
T1	1.028	0.786–1.345	0.839	0.819	0.586–1.146	0.244
T2	1.388	1.045–1.844	0.024	0.835	0.577–1.207	0.337
T3	1.545	1.145–2.084	0.004	1.159	0.769–1.748	0.480
T4	3.340	2.377–4.693	<0.001	1.390	0.898–2.153	0.140
Stage-N			<0.001			0.012
N0	1.000			1.000		
N1	1.618	1.222–2.143	<0.001	0.926	0.660–1.299	0.655
N2	2.313	1.676–3.192	<0.001	1.534	1.071–2.197	0.020
N3	3.369	1.668–6.802	<0.001	2.011	0.923–4.380	0.079
Stage-M			<0.001			<0.001
M0	1.000			1.000		
M1	3.748	2.849–4.931	<0.001	2.523	1.800-3.537	<0.001
Stage-T of RC			0.005			0.094
Ta	1.000			1.000		
T1	0.847	0.610–1.176	0.322	0.841	0.593–1.192	0.330
T2	1.036	0.730–1.471	0.843	1.106	0.740–1.653	0.623
T3	1.132	0.841–1.524	0.415	1.009	0.682–1.495	0.963
T4	2.028	1.295–3.178	0.002	1.917	1.145–3.211	0.013
Stage-N of RC			0.017			0.721
N0	1.000			1.000		
N1	1.187	0.942–1.496	0.146	0.975	0.753–1.261	0.844
N2	1.848	1.324–2.578	<0.0001	1.314	0.894–1.932	0.164
Stage-M of RC			<0.001			<0.001
M0	1.000			1.000		
M1	3.828	2.747–5.336	<0.001	3.113	2.144–4.521	<0.001
SPMs surgical history			<0.001			<0.001
Yes	1.000			1.000		
No	2.403	1.974–2.924	<0.001	2.056	1.552–2.725	<0.001
SPMs radiation record			<0.001			0.129
Yes	1.000			1.000		
No	0.707	0.577–0.866	<0.001	1.217	0.949–1.560	0.122
SPMs chemotherapy record			<0.001			0.177
Yes	1.000			1.000		
No	0.581	0.482-0.700	<0.001	0.874	0.682–1.120	0.287
SPMs tumor size(cm)			<0.001			0.140
0–3	1.000			1.000		
3–5	1.616	1.262–2.069	<0.001	1.377	1.032–1.839	0.030
5–10	2.005	1.544–2.602	<0.001	1.365	1.007–1.850	0.045
≥ 10	0.965	0.613–1.520	0.879	0.792	0.472–1.330	0.378

Abbreviations: SPMs: second primary malignancies; RC: rectal cancer

### Performance and validation of the nomogram

**Fig. 4:**
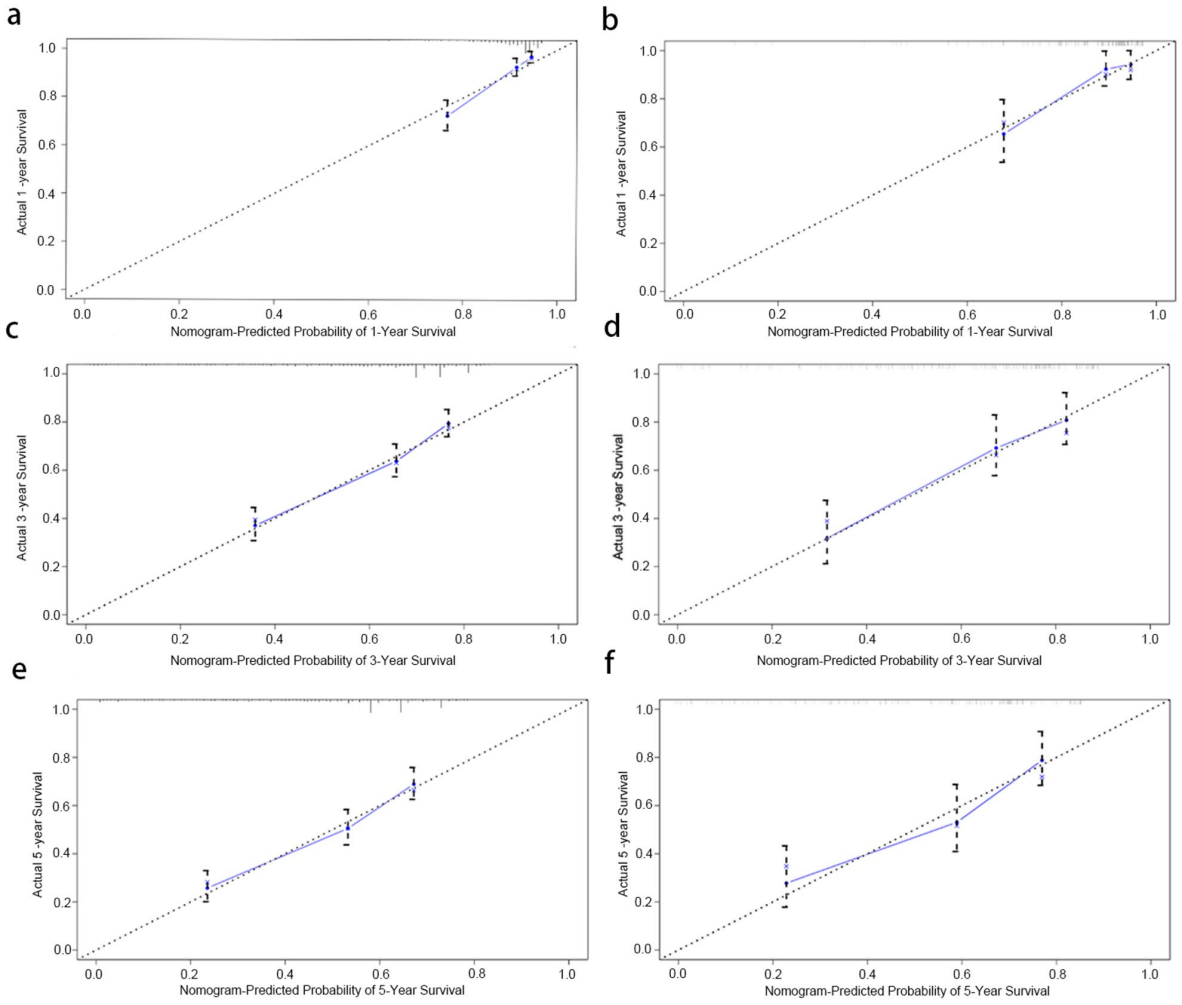
The calibration curve to evaluate the 1-year(a),3-year (**c**) and 5-year (**e**) survival for second primary malignancy (SPM) patients in the training set; The calibration curve to evaluate the 1-year(b),3-year (**d**) and 5-year (**f**)survival for SPM patients in the validation set

**Fig. 5:**
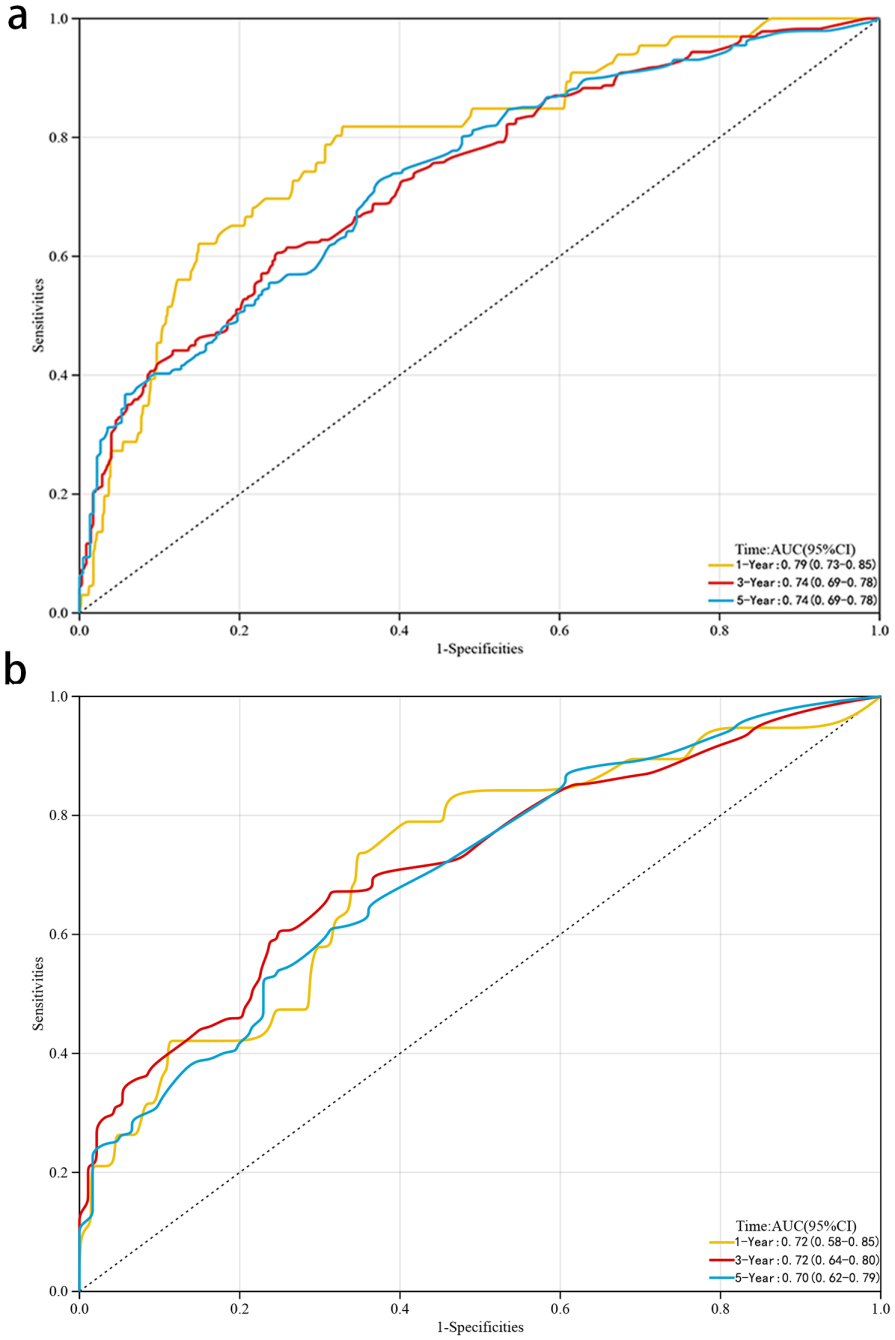
The ROC of 1-, 3-, and 5-year of the training **(a)** and validation **(b)** sets

To assessed the discriminative potential of the constructed nomogram in this study, C-index in the training set 0.691 (95% CI, 0.662–0.720) and validation set 0.731 (95% CI, 0.676–0.786) was calculated, indicating that the nomogram has moderate accuracy. To assessed the correctness of our model, calibration plots were utilized to verify the consistency of our prediction and actual outcomes. The 1-, 3-, and 5-year 0 S calibration curves fit well with the 45° diagonal, indicating an excellent performance of the nomogram (Fig. 4). Meanwhile, the time-dependent ROC curves at 1-,3-and 5-year illustrated that the nomogram was more accurate in predicting OS prognosis in the training set 0.79 (95%,0.73–0.85),0.74 (95, 0.69–0.78) and 0.74 (95%,0.69–0.78), and validation set 0.72 (95%CI,0.58–0.85),0.72 (95%CI,0.64–0.80), and 0.70 (95%,0.62–0.79) (Fig. 5), respectively.

**Fig. 6:**
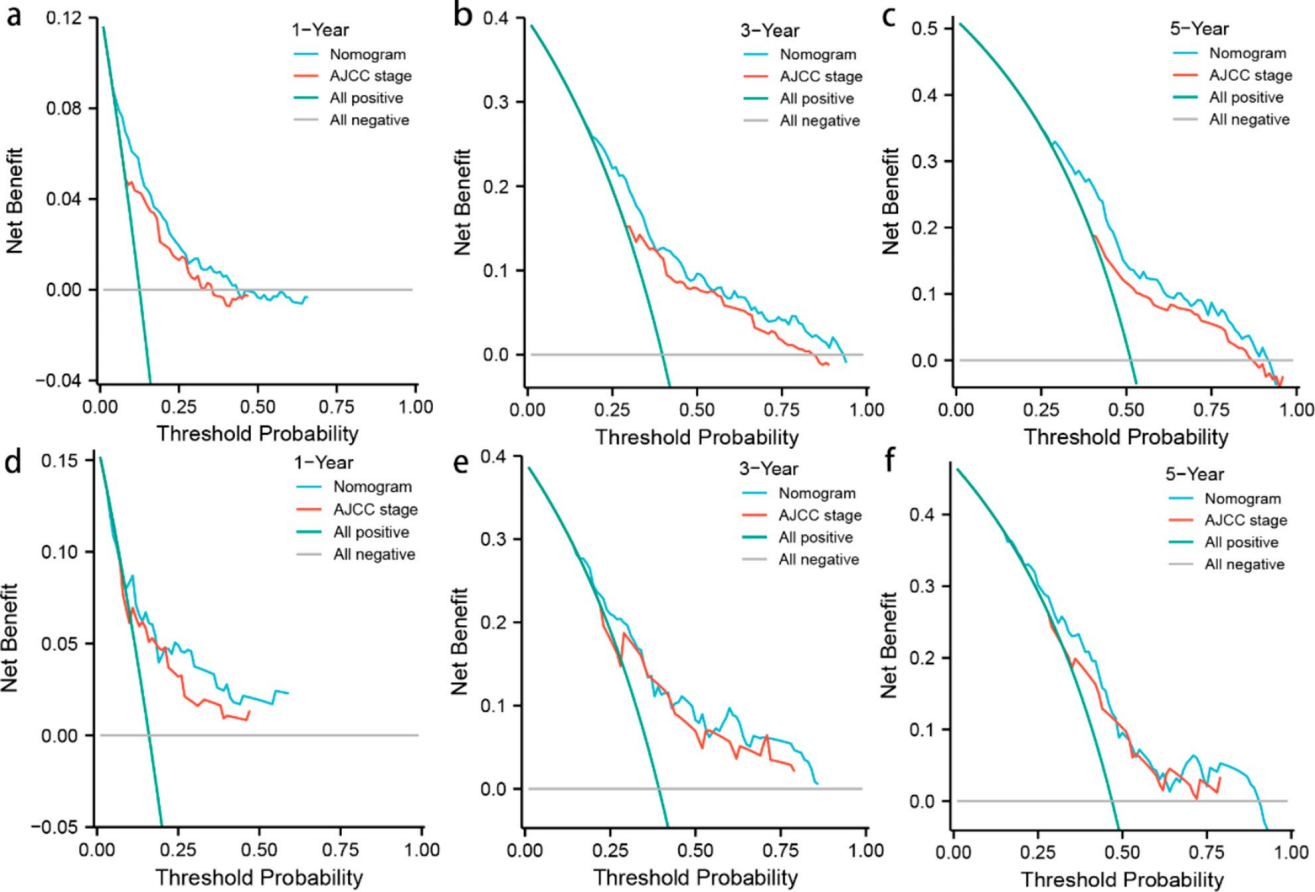
DCA curves of the nomogram and AJCC TNM staging system for predicting 1-,3- and 5-year OS in the training set (**a, b, c**), the internal validation set (**d, e, f**)

As shown in Fig. 6, DCA curves showed that the nomogram could more accurately forecast the likelihood of OS occurring after 1, 3, and 5 years, which, in both groups, may offer greater net clinical advantages than the AJCC stage model. Furthermore, we utilized the NRI and IDI to compare the accuracy of the nomogram with the usual AJCC staging system (Table 4). In the training set, the NRI for 1-3- and 5-year OS were 0.247(95%CI 0.022–0.503), 0.445(95%CI 0.363–0.689) and 0.445(95%CI 0.363–0.689), while the NRI for 1-3- and 5-year OS were 0.247(95%, CI 0.024–0.506), 0.445(95%, CI 0.299–0.682) and 0.075(95%CI 0.400–0.720) in the validation set. Additionally, the INI for 1-3- and 5-year OS were 0.030(*P*<0.001),0.072(*P*<0.001), and 0.080(*P*<0.001) in the training set, and 0.068(*P*<0.001),0.131(*P*<0.001) and 0.141*(P*<0.001) in the validation set. The NRI and IDI results demonstrated that the accuracy of the nomogram to predict OS is much superior than the usual AJCC staging system.

**Table 4:** NRI and IDI of the nomogram and the traditional AJCC staging system in OS prediction for RC patients

	NRI			IDI		
	1-Year	3-Year	5-Year	1-Year	3-Year	5-Year
Training set(N = 585)						
Estimate	0.247	0.445	0.508	0.030	0.072	0.080
95%CI	0.022–0.503	0.363–0.689	0.385–0.682			
P value				<0.001	<0.001	<0.001
Validation set(N = 156)						
Estimate	0.247	0.445	0.508	0.068	0.131	0.141
95%CI	0.024–0.506	0.299–0.682	0.400–0.720			
P value				<0.001	<0.001	<0.001

**Fig. 7:**
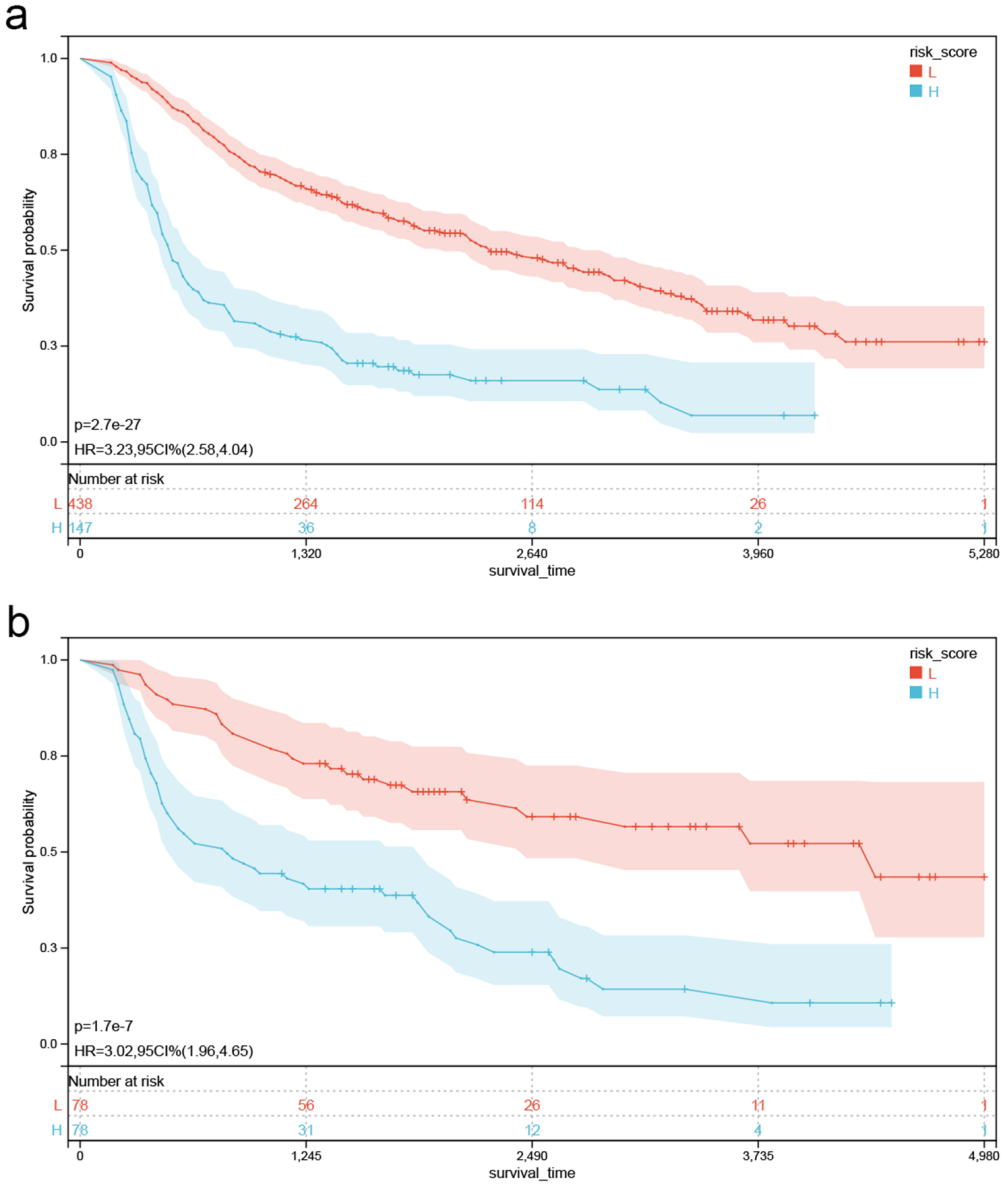
Kaplan-Meier curves of OS for risk score in the training set(*P*<0.001) **(a)**, the internal validation set(*P*<0.001)**(b)**

Finally, a risk score for each patient was calculated by nomogram with an establishment of risk stratification (Fig. 7). In both the training (Fig. 7A) and validation (Fig. 7B) sets, the Kaplan-Meier survival curves displayed remarkable statistical difference between high and low-risk individuals (p<0.001).

## Discussion

As the incidence of SPMs increased significantly, recent developments in SPMs had heightened the need for research on the monitoring, prognosis, and treatment decisions for clinical and public health [20, 21]. To investigated the prognosis of SPMs following RC, 9 parameters including the site, age, stage TNM, stage M of RC, SPMs surgical history, radiotherapy records, chemotherapy records, and tumor size were analyzed, which were applied to create a new nomogram that forecasts the survival rate of SPM patients. Taken together, our research showed that nomograph is superior to the AJCC staging system in predicting the probability of OS after 1 year, 3 years, and 5 years in the training set and validation set.

In reviewing the literature, Du et al. [22] reported that the three most prevalent sites of SPMs were neoplasms of colorectum (SIR 1.59, 95%CI 1.38–1.83), corpus uteri (SIR 2.11, 95%CI 1.62–2.76), and small intestine (SIR 4.00, 95%CI 2.91–5.49) in recently mete-analysis. Xu et al. [23] showed that Patients with RC were more likely to develop malignant tumors in the thyroid, uterine body, colon, rectum, lung/ bronchus. The same as our research results showed that the three most popular sites for SPMs were the Lung and Bronchus (18.35%), Urinary Bladder (15.11%), and Breast (11.20%). Therefore, it is of great significance to regular and long-term monitoring of the Lung and Bronchus, Urinary Bladder, and Rectum, which was necessary for RC patients at high risk.

Among the 9 parameters included in our nomogram, Age was recognized important risk contributor for SPM patients [24, 25]. Liu et al. [26] reported that Age (50–59:HR 0.958, 95%CI 0.842 − 0.091; 60–100:HR 1.557, 95%1.370–1.747; 18–49 as a reference) by multivariate analysis were all correlated with OS (*P*<0.001). Similarly, Li et al. [27] noted that Age (≥ 73:HR 1.482,95%CI 1.048–2.152; <73 as a reference) by multivariate analysis were all correlated with OS(*P* = 0.045). After dividing age into four age groups to better explore the relationship between age and overall survival, the results indicate that Age (60–69:HR1.422,95%CI1.074-1.883;70–79:HR 1.713,95%1.297–2.263; ≥80:HR 2.801,95%11.763–4.450; <60 as a reference) by multivariate analysis were all correlated with OS (*P* < 0.001). The degradation of the physical state, terrible treatment sensitivity, and the worsening cancer stage in elderly patients may all be contributing factors to these results.

Likewise, multivariate analysis in our study revealed that N stage (N1:HR 0.926, 95%CI 0.660–1.299; N2:1.534 95%CI 1.071–2.197;N3:HR 2.011,95%CI 0.923–4.380; N0 as a reference) for SPM patients had statistically significant OS rates(*P* = 0.012). This is consistent with those the findings of previous work that the N stage was one of the most significant contributions to OS [28, 29]. This view is supported by Park et al. [30] who reported that patients had higher pathological N stage (N1:HR 1.182,95%CI 1.191–1.845, *P*<0.001; N2:2.344 95%CI 1.779–3.289, *P*<0.001; N0 as a reference) significantly associated with OS, suggesting that surveillance was more frequent. As noted by Song et al [31], the N stage was considered as a potential predictor by LASSO, whose classification contributes most to the prognosis of survival in the nomogram they constructed.

Nomogram as a suitable scoring tool for clinical research, it could integrate the effects of various prognostic factors and present the results intuitively. Compared with the current AJCC sixth edition, the nomogram we created demonstrates a noticeably stronger capacity for risk stratification of RC SPM patients. Meanwhile, it is straightforward to gather nine prognostic factors on SPM patients, match that data with the nomogram we created, and calculate the corresponding scores. We could convenient to obtain the 1-, 3-, and 5-year OS by adding and matching the nomogram. The nomogram could help patients’ contributions to information on survival, clinical decision-making guidance, and treatment allocation. For those patients at high risk, they need active therapeutic and close monitoring to improve their overall survival.

Several questions still remain unanswered at present. First, although this study is a retrospective study and strictly complies with the inclusion and exclusion criteria, potential selection bias may have occurred. Secondly, Due to the lack of data relating to chemotherapy protocols and dose, it is not possible to evaluate the effects of different protocols and dose on the onset of secondary cancer. Finally, although our predictive model performs well through internal validation, additional external validation with other populations is still required.

## Conclusions

In summary, this study was conducted to describe the clinical characteristics of SPMs in RC survivors and 9 clinical parameters are chosen to create a nomogram to forecast the 1-, 3-, and 5-year OS of SPM patients. It was also shown that the model prediction for OS in SPM patients was superior to the SEER historic stage with RC. Taken together, our findings might provide clinical prognostic guidelines for SPM patients, whose actual efficiency should be further improved through larger research further.

### Electronic supplementary material

Below is the link to the electronic supplementary material.

Supplementary Material 1

Supplementary Material 2

## Data Availability

Original data are available from the corresponding author(extract the data in the SEER database: (http://seer.cancer.gov).

## Abbreviations

RC: Rectal Cancer
OS: Overall Survival
ROC: Receiver Operating Characteristic
IPC: Initial Primary Cancer
LNR: Lymph Node Removed
NRI: Nutrition Risk Index
IDI: Integrated Discrimination Improvement
DCA: Decision Curve Analysis
SEER: Surveillance, Epidemiology, and End Results
