# Supplementary material for: Risk prediction of second primary malignancies in patients after rectal cancer: analysis based on SEER Program
Source: BMC Gastroenterol. 2023 Oct 12;23:354. doi: 10.1186/s12876-023-02974-2 (PMC10568885; doi:10.1186/s12876-023-02974-2)
Supplement: Supplementary file 1 — Supplementary Material 1 [file 12876_2023_2974_MOESM1_ESM.doc]

**Table S1.** Site of SPMs after RC that the remaining**.**

| **Site of SPMs** | **N** | **%** |
| --- | --- | --- |
| Thyroid | 9 | 1.21% |
| Transverse Colon | 9 | 1.21% |
| Penis | 6 | 0.81% |
| Testis | 6 | 0.81% |
| Hypopharynx | 6 | 0.81% |
| Descending Colon | 5 | 0.67% |
| Vulva | 5 | 0.67% |
| Gum and Other Mouth | 5 | 0.67% |
| Small Intestine | 5 | 0.67% |
| Appendix | 4 | 0.54% |
| Salivary Gland | 4 | 0.54% |
| Hepatic Flexure | 4 | 0.54% |
| Bones and Joints | 4 | 0.54% |
| Nasopharynx | 4 | 0.54% |
| Cervix Uteri | 4 | 0.54% |
| Oropharynx | 3 | 0.40% |
| Retroperitoneum | 3 | 0.40% |
| Rectosigmoid Junction | 3 | 0.40% |
| Splenic Flexure | 3 | 0.40% |
| Mesothelioma | 3 | 0.40% |
| Peritoneum, Omentum and Mesentery | 2 | 0.27% |
| Vagina | 2 | 0.27% |
| Large Intestine, NOS | 2 | 0.27% |
| Liver | 2 | 0.27% |
| NHL - Nodal | 2 | 0.27% |
| Gallbladder | 2 | 0.27% |
| Other Endocrine including Thymus | 2 | 0.27% |
| Myeloma | 1 | 0.13% |
| Miscellaneous | 1 | 0.13% |
| Hodgkin - Extranodal | 1 | 0.13% |
| Floor of Mouth | 1 | 0.13% |
| Other Urinary Organs | 1 | 0.13% |
| Nose, Nasal Cavity and Middle Ear | 1 | 0.13% |
| Other Non-Epithelial Skin | 1 | 0.13% |
| Lip | 1 | 0.13% |
| Uterus, NOS | 1 | 0.13% |
| Other Digestive Organs | 1 | 0.13% |
| Other Oral Cavity and Pharynx | 1 | 0.13% |
| Other Female Genital Organs | 1 | 0.13% |

Abbreviations: SPMs: second primary malignancies; RC: rectal cancer.
