# Supplementary material for: Risk prediction of second primary malignancies in patients after rectal cancer: analysis based on SEER Program
Source: BMC Gastroenterol. 2023 Oct 12;23:354. doi: 10.1186/s12876-023-02974-2 (PMC10568885; doi:10.1186/s12876-023-02974-2)
Supplement: Supplementary file 2 — Supplementary Material 2 [file 12876_2023_2974_MOESM2_ESM.doc]

**Table S2.** Histology of SPMs after RC that the remaining**.**

| **Histology of SPMs** | **N** | **%** |
| --- | --- | --- |
| unspecified neoplasms | 2 | 0.27% |
| mucoepidermoid neoplasms | 2 | 0.27% |
| thymic epithelial neoplasms | 2 | 0.27% |
| plasma cell tumors | 1 | 0.13% |
| neopl of histiocytes and accessory lymphoid cells | 1 | 0.13% |
| hodgkin lymphomas | 1 | 0.13% |
| adnexal and skin appendage neoplasms | 1 | 0.13% |
| blood vessel tumors | 1 | 0.13% |
| lymphoid leukemias | 1 | 0.13% |

Abbreviations: SPMs: second primary malignancies; RC: rectal cancer.
